# Does Mortality Vary between Asian Subgroups in New Zealand: An Application of Hierarchical Bayesian Modelling

**DOI:** 10.1371/journal.pone.0105141

**Published:** 2014-08-20

**Authors:** Santosh Jatrana, Ken Richardson, Tony Blakely, Saira Dayal

**Affiliations:** 1 Alfred Deakin Research Institute, Deakin University Waterfront Campus, Geelong, Victoria, Australia; 2 Department of Public Health, Wellington School of Medicine and Health Sciences, University of Otago, Newtown, Wellington, New Zealand; Geisel School of Medicine at Dartmouth College, United States of America

## Abstract

The aim of this paper was to see whether all-cause and cause-specific mortality rates vary between Asian ethnic subgroups, and whether overseas born Asian subgroup mortality rate ratios varied by nativity and duration of residence. We used hierarchical Bayesian methods to allow for sparse data in the analysis of linked census-mortality data for 25–75 year old New Zealanders. We found directly standardised posterior all-cause and cardiovascular mortality rates were highest for the Indian ethnic group, significantly so when compared with those of Chinese ethnicity. In contrast, cancer mortality rates were lowest for ethnic Indians. Asian overseas born subgroups have about 70% of the mortality rate of their New Zealand born Asian counterparts, a result that showed little variation by Asian subgroup or cause of death. Within the overseas born population, all-cause mortality rates for migrants living 0–9 years in New Zealand were about 60% of the mortality rate of those living more than 25 years in New Zealand regardless of ethnicity. The corresponding figure for cardiovascular mortality rates was 50%. However, while Chinese cancer mortality rates increased with duration of residence, Indian and Other Asian cancer mortality rates did not. Future research on the mechanisms of worsening of health with increased time spent in the host country is required to improve the understanding of the process, and would assist the policy-makers and health planners.

## Introduction

New Zealand (NZ) has one of the highest proportions of overseas born people in the Western World (25% in 2013), compared with Australia (22%), Canada (18%), the United States (11%) and the UK (10%). Ethnic Asians make up 12% of the total New Zealand population and are the fastest growing ethnic group in New Zealand [1], [2]. From 1991 to 2001 the Asian population increased by 140%, the highest growth of any NZ ethnic group. Projections indicate that by the year 2021 there will be an estimated 670,000 Asians in NZ compared to 270,000 today, increasing the proportion of NZ’s population that is ethnically Asian from 10% in 2006 to 16% in 2026 [3]. The projected increase in the Asian population share is largely due to immigration and amounts to a net inflow of about 250,000 migrants over the 20-year period under a standard set of projection assumptions. Not only has the Asian proportion of the population increased over time but it became more heterogeneous. The largest NZ Asian subgroup is Chinese, followed by Indians, Filipinos, and Koreans [2]. The growing size and diversity of the NZ Asian population may have important implications for health needs and for planning and delivering health services.

Despite the significant increase of the Asian population and their rapid growth relative to other ethnic groups, there have been rather few investigations of Asian health and mortality at the national level [4], [5]. Much research on health inequalities in NZ is based on studies of the three major ethnic groups: Māori (the indigenous people of NZ), Pacific, and the majority non-Māori non-Pacific populations [6], [7]. Moreover, immigrant status (nativity) as a source of ethnic variations in health has not received enough attention, despite the fact that understanding health disparities in NZ is broadly accepted as an important public health objective [8], [9]. Furthermore, studies of NZ Asian mortality have not explored the diversity of the Asian population e.g., by combining all people of Asian origin [10]. Other research has combined age, sex and cohort data of major sub-ethnic groups to increase the number of people and deaths in each subgroup, but have then been unable to consider the association of immigration-related factors (e.g., nativity or duration of residence in New Zealand) with Asian subgroup mortality [4], [5]. While Hajat et al (2010) considered nativity and duration of residence (DoR) in the estimation of ethnic-specific mortality, subgroup estimates were not possible given the small numbers of deaths in some cells when stratified by country of birth and years of residence [10]. However, not accounting for the heterogeneity of Asian sub-populations can obscure the significant differences in health outcomes between ethnic subgroups and carries the risk that areas of greater health need may also be obscured by inappropriate aggregation [5], [11]–[14].

A breakdown of mortality inequalities by specific Asian groupings (e.g., Indians, Chinese, and Other Asians) is nevertheless challenging due to small numbers of deaths in specific strata (the “small-cell” problem) and a lack of data linking health outcomes to migration status [15]. Accordingly, the objective of the present research is to examine disparities in all-cause and cause-specific mortality of Asian subgroups (Chinese, Indians and Other Asians), with special attention to the effect of nativity (i.e., whether individuals are overseas born (OSB) or New Zealand born (NZB)), and to time spent in New Zealand since immigration. This study overcomes potential numerator-denominator bias when studying Asian subgroup mortality by using linked census-mortality cohorts for 1996–1999 and 2001–06 from the New Zealand Census-Mortality Study (NZCMS), and the problem of small numbers by using a hierarchical Bayesian modelling technique - an approach that is applicable to analyses of other migrant groups for which sparse data is problematic [16]. To our knowledge this study is the first assessment of variation in all-cause and cause-specific across specific Asian adult sub-populations accounting for the effects of nativity and duration of residence in New Zealand. It addresses the following specific research questions:

1. What are the all-cause and cause-specific (cancer and cardiovascular (CVD)) mortality rates for large Asian subgroups (Chinese, Indians, and Other Asians)?
2. Do foreign born Asian subgroups have an all-cause and cause-specific mortality rate advantage relative to their NZB counterparts of the same ethnicity? Does this relationship operate differently for different Asian subgroups?
3. If OSB Asian subgroups have an all-cause or cause-specific mortality rate advantage, does it decline as duration of residence increases and is the decline the same for all OSB Asian subgroups?

Migration has long been linked to health. For example, growing evidence from research on immigrant health in North America and Canada [13], [14], [17]–[21], Europe [22]–[24], and Australia [25]–[27] has shown that immigrants to a new country often exhibit similar or better health upon arrival in the destination country than their native born counterparts, despite (often) lower socioeconomic status that might suggest poorer health profiles. This phenomenon is often referred to as the “immigrant health paradox” or “Hispanic paradox”. Over time however, their health declines below that of new migrants or to the level of their native born counterparts [17], [19]–[21].

The explanations offered for the initial advantaged health status of immigrants focus on the “healthy immigrant effect”, which assumes only those with good health are selected for migration, and the “unhealthy emigrant effect”, which supposes that some migrants who become (chronically) ill return to their ‘home’ (source) country to die and are thus not counted in the mortality rate of the destination country [28], [29]. While selection of healthy persons from the source country could be due to the requirement that potential migrants undergo medical screening (direct selection), or from immigration policies favouring tertiary education, occupational skills and wealth (indirect selection), many factors could affect return migration: for example, distance from the ‘home’ country, ease of return, eligibility for superannuation and access to and quality of health care in the home country. However, the unhealthy emigrant effect is less likely among Asian groups and we return to this point in the discussion.

Explanations for declining health with increased duration of residence in the host country concentrate on the increased risk-taking behaviour, such as poor diet, taking up alcohol and smoking as a result of acculturation, loss of family support and cultural orientation [13], [20]. In reality, acculturation can have a positive or a negative impact on health depending on the style of acculturation [30], differences in health between the source and destination countries, and the strength of health selection for the particular migrant population. For example, improvements in health care utilisation among Asians in New Zealand were recently documented [31].

Discrimination has also been proposed as an explanation for eroding the health-protective effect with longer residency in the host nation [32], [33]. Discrimination is a determinant of an individual’s state of health, which in turn is linked to social structure and hierarchy, socioeconomic class, gender and ethnic group [34]. Discrimination can directly cause adverse effects on health, or it can impact health through its relation to access to health care services [35], [36]. All migrants are at some risk of experiencing discrimination because of their overseas born status, but characteristics such as visible minority status may place such migrant communities at additional risk [36]. Thus, the health status of the migrant population may be improved or disadvantaged relative to their counterfactual health status (i.e., had they not migrated) as a result of multiple processes working in different directions.

A full explanation for health inequalities within and between OSB Asian subgroups and NZB Asian subgroups would require consideration of several migration-related processes (e.g., selection, acculturation and discrimination) as well as New Zealand-specific factors (e.g. differential socioeconomic position by ethnic groups, biological susceptibility to some diseases). However, focusing just on migration, it is difficult to estimate the independent effects of health selection for migration and return migration, the waning of the consequent health benefits (should they exist) over time, and the health impacts of acculturation, as well as the experience of interpersonal and systemic racial discrimination in the destination country. Nevertheless, by examining whether mortality rates of OSB Asian subgroups upon arrival in New Zealand are different from mortality rates of NZB counterparts and, if a significant difference exists, by determining whether the effect changes as duration of residence increases, we can investigate the existence of a ‘healthy immigrant effect’ - an effect that states that migrants are in better health upon arrival in the host country than their NZB counterparts but that this advantage erodes over time. Duration of residence (0–9, 10–24, and 25 years or longer) was used here as a proxy measure to capture the net result of the waning of health selection effects, the effects of acculturation, and exposure to racial discrimination on the health of Asian subgroup migrant populations. We do not attempt to separately distinguish the effects of age, age-at-migration, and duration of residence because of the linear relationship between them.

### Asian Migration to New Zealand

More formalised immigration to New Zealand for non-Māori New Zealanders began in 1840. Migrants were almost exclusively from Britain. The first Asians to arrive in numbers were Chinese men in 1866, invited due to their reputation as diligent workers in the gold fields. Approximately 1,000 arrived in the first year and by 1881 numbers exceeded 5,000. Immigrants from India arrived initially only in small numbers, with only 181 recorded in the 1916 census [37], [38].

However, concern from the white majority over the growing Asian population resulted in restrictions on immigration. In 1881 the number of Chinese migrants was restricted and a tax on arrival introduced [38]. As British subjects, the restriction of Indians was more difficult. An attempt was made by requiring Indian migrants to write and read passages of English, but the development of ‘cramming schools’ in Fiji and on passenger boats, and the entry of many boys as ‘sons’ of current residents meant that by 1945 there were over 2,000 Indians in New Zealand [39]. Additionally, New Zealand’s alliance with China during the Second World War saw some restrictions lifted. By 1966 the Chinese population had grown to over 10,000 with around 75% being New Zealand born [40].

A significant change in migration to NZ followed the introduction of the 1987 Immigration Act. The Act signalled a move away from preferring migrants from ‘traditional source’ countries to an ‘internationalist non-discriminatory’ policy aimed at encouraging economic growth. Migration from Asia increased significantly with large numbers arriving from China, Hong Kong, Korea, Japan, the Philippines, India and Sri Lanka; as well as Indians arriving from Fiji following the military coups. Many migrants were wealthy and highly skilled, entering under the ‘skilled’ and ‘business’ migrant categories [40]–[42]. Subsequent changes to immigration policy have resulted in fewer, but still significant numbers of Asian migrants arriving annually. By the 2013 census, the Asian population in New Zealand was ethnically, socially and socio-economically diverse and comprised 11.8% of the total population.

## Research Design and Methods

The New Zealand Census-Mortality Study, on which the present work is based, was approved by the Central Regional Ethics Committee, Ministry of Health, Wellington, New Zealand. Respondent information was anonymised before analysis, which is a Statistics New Zealand requirement under the NZ Statistics Act 1975. This study used the 1996–1999 and 2001–2004 NZCMS cohorts in which census records have been anonymously and probabilistically linked to mortality records. The NZCMS methodology has been described in detail elsewhere [43], [44]. Briefly, eligible 1996–99 and 2001–04 death registration records were linked to the national 1996 and 2001 census. Not all mortality records were linked to a corresponding census record, and it was necessary to correct for any linkage bias and consequent underestimation of mortality rates. Weights were based on variables that predicted linkage in logistic regression analyses: age at census, sex, prioritised ethnicity, rurality, residential mobility of area unit, Territorial Authority, NZ deprivation index, months since census night at death, and cause of death. We excluded persons under age 25 to ensure all age strata were populated by migrants with long durations of residence. Those aged 75 and older are not represented in the 1996–1999 cohort and were excluded from this study.

For the OSB:NZB comparison, data were restricted to 617,427 person-years of follow-up for 25–74 year old Chinese, Indian, and Other Asian people with non-missing data on follow-up time and DoR. Within each of these three ethnicities, analyses were conducted on 40 strata formed by cross-classifying sex (dichotomous) by age (10 groups) by nativity (2 groups). For the comparison of OSB Asian mortality rates by DoR, 546,765 person-years of follow-up were available for analysis. Within each ethnicity 60 strata were constructed by cross-classifying sex by age (10 groups) by DoR (3 groups: 0–9, 10–24, and 25+ years).

The ethnicity variable was classified using a “total count” definition [45]. For example, all of the following people would be categorised as Chinese in this paper: self-identified Chinese only; self-identified Chinese and Indian; self-identified Chinese and NZ European; and self-identified Chinese and Māori. Using the total count method self-identified Chinese and Indian people in this example would be counted in both Chinese and Indian groups.

For both the OSB:NZB and DoR analyses, ten age-groups were used: 25–29, 30–34, 35–39, 40–44, 45–49, 50–54, 55–59, 60–64, 65–69, and 70–74 years, centred at 45–49 years, and scaled so that each unit increase in scaled age corresponds to an actual increase of 5 years. Thus the above age ranges are represented by their end-points which, after centring and scaling become (−5, −4, −3, −2, −1, 0, 1, 2, 3, 4, 5). To allow for the non-linear increase in mortality with age, a linear spline for age with knots at the 35–39 and 55–59 age groups was used.

The study design and linkage process reduces the potential for numerator-denominator bias i.e., non-comparability of country of birth and years of usual residence between census and mortality data [46]. As previously noted, to correct for linkage bias (i.e., variability in success linking mortality and census records of immigrants as compared to native born persons because of less complete or accurate record keeping), linkage weights have been developed for strata based on age, sex, ethnicity and small area deprivation. For example, if 20 out of 30 deaths for OSB Indian males aged 45–64 living in the north of New Zealand and in the most deprived neighbourhoods were linked back to their census record, then each of these linked records receive a weight of 1.5 (30/20), thus making analyses representative of all eligible deaths. Elsewhere, we have shown these linkage weights to be valid [47], although not specifically for Asian populations. Unit record NZCMS data available in the Statistics New Zealand Data Laboratory was stratified by the variables of interest (including ethnic subgroup), and the deaths and person-time (person-years at risk) in each stratum recorded. These aggregated data were confidentialised by Statistics New Zealand and “exported” for analysis.

Given the small number of deaths in some strata (e.g., at younger ages), we used hierarchical Bayesian regression modelling that allows pooling of information across strata (and therefore some smoothing of posterior mortality rates) by shrinkage towards a prior covariate structure. [48]. We extended HB methods used previously in the NZCMS [16], [49]. To allow comparison across ethnic groups, and other strata of interest (OSB/NZB, DoR), stratum-specific posterior mortality rates were estimated using the Bayesian package WinBugs. Comparison of ethnic-specific mortality rates (reported below as deaths per 100,000 person-years) was done by directly standardising stratum-specific posterior rates to the total Asian population, defined as a combination of the 1996–1999 and 2001–2004 cohorts, following the approach of Richardson, Jatrana et al. 2013 [49]. Rate ratios were then computed from standardised rates and used to summarise differences in ethnic- and cause-specific posterior mortality rate distributions in two ways. First, within each stratum of natality and cause of death, rate ratios of the Indian and Other Asian groups (relative to the Chinese group) were computed to determine the significance of differences in standardised posterior mortality rates within that stratum. The computation was repeated for each stratum of DoR within the OSB group. Second, ethnic- and cause-specific posterior rate ratios were computed for the 0–9 year and 10–24 year DoR groups relative to the 25+ year group within the OSB group to determine the significance of changes in posterior mortality rates by DoR. To robustly summarise posterior rates and rate ratio distributions we used sample medians as the main estimate of centrality, and 95% credible intervals to quantify the width of posterior distributions. Access to a large sample of posterior mortality rates is one of the advantages of our Bayesian approach, since more information about the distribution of the parameters of interest is available than in many non-Bayesian analyses. For example, if numerator and denominator posterior distributions in a rate ratio substantially overlap the rate ratio 95% credible interval will be roughly centred on unity, and we can be reasonably sure there is no significant difference between them. Conversely, if 95% credible intervals exclude unity, we can be reasonably sure that the differences are significant. However, if credible intervals include unity, but “only just”, estimates of the probability that the rate ratio is greater than (or less than) 1 can be made to assess the significance of overlap between numerator and denominator distributions. We have used this approach in the analysis below, noting as significantly different those numerator and denominator posterior distributions where less than 10% of posterior rate ratios are greater (or less than) 1.

Analyses were done using the R environment (http://www.r-project.org) for statistical computation version 2.13.0 available from the Comprehensive R archive Network (CRAN) website (http://cran.r-project.org) or SAS 8.2 (SAS Institute Inc., Cary, North Carolina). All HB analyses used WinBugs 1.4, available from (http://www.mrc-bsu.cam.ac.uk/bugs/), and the R2WinBUGS package version 2.1–16.

## Results

Total follow-up times and deaths (weighted) by age and sex for the aggregated all-Asian population are given in Table 1. The sparseness of data in this highly stratified dataset is highlighted by the occurrence of the number 6 in the table (the minimum publishable value permitted for counts under Statistics New Zealand’s confidentiality rules), despite the relatively wide age categories used for this table.

**Table 1:** Person time and deaths by sex by age and DoR, 2001**–**2004 cohort, ‘total’ definition of ethnicity, aggregated over the three Asian groups.

		OSB		DoR (0–9)		DoR (10–24)		DoR (25+)		NZB	
Sex	Age	Person Years	Deaths^1^	Person years	Deaths	Person years	Deaths	Person years	Deaths	Person years	Deaths
**M&F**	25–74	546765	996	380808	492	113862	201	52098	306	70659	165
	25–39	248388	123	195735	81	45306	39	7353	6	39435	39
	40–54	213744	243	137376	144	54339	60	22023	45	22206	39
	55–74	84636	630	47694	267	14217	105	22719	261	9018	84
**Males**	25–74	244224	588	166377	273	52356	120	25491	201	34641	111
	25–39	106797	60	82284	39	21027	21	3489	6	19314	30
	40–54	96357	144	61332	84	24465	39	10560	21	11013	21
	55–74	41070	384	22758	147	6867	54	11445	177	4308	63
**Females**	25–74	302544	405	214431	216	61503	81	26601	105	36018	51
	25–39	141588	60	113448	45	24279	18	3861	6	20118	9
	40–54	117384	99	76047	57	29877	18	11463	21	11196	18
	55–74	43566	246	24939	114	7353	48	11277	87	4710	24

All counts are random rounded to a near multiple of three with a minimum cell size of 6, as per Statistics New Zealand protocols. Person years are weighted person years.

1 Weighted deaths.

As shown in Table 2, standardised all-cause mortality rates were highest for the NZB Indian ethnic group (390.4, 95% CI (308.4, 485.9)), significantly so when compared with those NZB of Chinese ethnicity (rate ratio 1.39, 95% CI (1.10, 1.73)). A similar result was found for the OSB Indian ethnic group (mortality rate 276.8, 95% CI (240.2, 318.1; rate ratio 1.46 95% CI (1.24, 1.73)). For all ethnicities, posterior all-cause mortality rates for the OSB were significantly less than for the NZB with median rate ratios between 0.63 and 0.71 i.e., overseas born Asian people have about two thirds of the mortality rate of NZB Asian people.

**Table 2:** Posterior median standardised mortality rates (95% CIs) and standardised rate ratios (95% CIs) by natality and cause of death.

Ethnicity	Rates		Rate ratios		
	NZB	OSB	NZB^1^	OSB^1^	OSB to NZB
**All-cause**					
Chinese	281.2 (230.7,338.7)	189.2 (167.8,213.2)	1	1	0.67 (0.55,0.83)
Indian	390.4 (308.4,485.9)	276.8 (240.2,318.1)	1.39 (1.10,1.73)	1.46 (1.24,1.73)	0.71 (0.57,0.90)
Other Asian	305.4 (233.5,397.1)	190.9 (158.8,224.0)	1.09 (0.85,1.38)	1.01 (0.82,1.22)	0.63 (0.48,0.79)
**Cancer**					
Chinese	107.3 (76.5,148.9)	70.5 (57.2,85.5)	1	1	0.66 (0.46,0.94)
Indian	73.7 (47.6,112.1)	51.5 (38.3,68.4)	0.69 (0.45,1.01)	0.73 (0.52,1.01)	0.70 (0.47,1.07)
Other Asian	106.0 (65.7,163.4)	71.5 (53.4,92.8)	0.99 (0.65,1.46)	1.01 (0.74,1.38)	0.67 (0.45,1.03)
**CVD**					
Chinese	88.6 (63.2,123.8)	60.0 (48.6,74.6)	1	1	0.68 (0.48,0.97)
Indian	197.2 (134.9,278.0)	136.6 (110.2,166.4)	2.22 (1.53,3.12)	2.27 (1.73,2.94)	0.69 (0.49,1.01)
Other Asian	94.0 (58.7,148.8)	57.5 (39.9,78.6)	1.06 (0.68,1.64)	0.96 (0.64,1.36)	0.61 (0.39,0.91)

1 Chinese reference (same natality).

Cancer mortality rates for people of Indian ethnicity were lower than other ethnicities (73.7, 95% CI (47.6, 112.1) and 51.5, 95% (38.3, 68.4) for the NZB and OSB respectively with median rate ratios relative to the Chinese ethnic group of 0.69 and 0.73 for the NZB and OSB respectively. The differences in cancer mortality rates between Indian and Chinese ethnic groups were significant even though credible intervals just included unity since only 3% of posterior rate ratios were greater than 1 for both OSB and NZB groups. In contrast, CVD mortality rates for the Indian ethnic group were significantly higher than for the other ethnicities: 197.2, 95% CI (134.9, 278.0) for the NZB and 136.6, 95% CI (110.2, 166.4) for the OSB, with median rate ratios (relative to the Chinese group) around 2.2.

As with all-cause mortality, cancer and CVD mortality rates were significantly smaller for the OSB than for the NZB for all ethnic groups with median OSB:NZB rate ratios between 0.61 and 0.70. Once again, some credible intervals for these rate ratios included the null but only just e.g., only 3% of the Indian OSB:NZB CVD rate ratios were greater than 1.

Results for standardised mortality rates by ethnicity and DoR within the OSB population are shown in Table 3. For all-cause mortality, rates for people of Indian ethnicity were higher than for other ethnicities at each DoR e.g., 221.3, 95% CI (182.9, 265.8) for those resident in NZ for 0–9 years, with a rate ratio (relative to the Chinese group with the same DoR) of 1.55, 95% CI (1.25, 1.95). For all ethnicities, all-cause mortality rates for all ethnic groups living in NZ for less than 10 years were significantly less than those living in NZ for 25 years or more with median rate ratios between 0.52 and 0.63. For people living in NZ between 10 and 24 years median posterior all-cause rate ratios (relative to those living in NZ for 25 years or more) were larger (around 0.8), but still reasonably significant with only 7% (Chinese), 6% (Indian) and 7% (Other Asian) exceeding 1. These results are consistent with all-cause mortality increasing steadily with DoR for all ethnic groups.

**Table 3:** Posterior median standardised mortality rates (95% CIs) and standardised rate ratios (95% CIs) by DoR and cause of death.

Ethnicity	Rates			Rate ratios				
	DoR (0–9)	DoR (10–24)	DoR (25+)	DoR (0–9)^1^	DoR (10–24)^1^	DoR (25+)^1^	DoR (0–9)^2^	DoR (10–24)^2^
All-cause								
Chinese	142.2 (120.2,167.3)	227.2 (179.5,286.7)	276.4 (231.8,328.1)	1	1	1	0.52 (0.41,0.64)	0.82 (0.63,1.08)
Indian	221.3 (182.9,265.8)	289.4 (222.5,371.7)	360.2 (297.3,432.1)	1.55 (1.25,1.95)	1.27 (0.97,1.67)	1.30 (1.04,1.61)	0.62 (0.49,0.79)	0.80 (0.60,1.06)
Other Asian	167.7 (137.3,205.5)	217.6 (166.5,281.5)	267.5 (204.7,343.6)	1.18 (0.93,1.50)	0.96 (0.71,1.27)	0.97 (0.73,1.25)	0.63 (0.47,0.84)	0.81 (0.60,1.11)
Cancer								
Chinese	60.1 (45.5,77.8)	77.5 (50.5,118.0)	81.6 (58.3,113.5)	1	1	1	0.73 (0.49,1.10)	0.95 (0.58,1.56)
Indian	52.0 (35.3,74.8)	52.7 (31.4,85.9)	50.2 (31.5,75.7)	0.86 (0.56,1.33)	0.68 (0.40,1.14)	0.61 (0.38,0.97)	1.04 (0.64,1.70)	1.05 (0.59,1.90)
Other Asian	72.0 (51.5,99.5)	67.6 (41.4,106.0)	76.3 (46.3,120.8)	1.20 (0.81,1.79)	0.87 (0.51,1.45)	0.94 (0.56,1.50)	0.95 (0.57,1.62)	0.89 (0.49,1.58)
CVD								
Chinese	41.5 (29.6,56.5)	83.6 (54.2,128.6)	89.4 (65.8,120.0)	1	1	1	0.46 (0.31,0.69)	0.94 (0.57,1.53)
Indian	92.3 (65.6,126.1)	136.1 (81.8,216.3)	192.2 (145.7,249.2)	2.22 (1.46,3.40)	1.63 (0.91,2.74)	2.15 (1.50,3.10)	0.48 (0.32,0.71)	0.71 (0.41,1.16)
Other Asian	40.6 (25.9,61.6)	71.6 (41.2,116.5)	84.3 (50.4,133.2)	0.98 (0.59,1.59)	0.85 (0.46,1.50)	0.94 (0.56,1.55)	0.48 (0.28,0.84)	0.84 (0.46,1.57)

1 Chinese reference (same DoR).

2 DoR (25+) reference.

On the other hand, there was little evidence that cancer mortality rates for the Indian and Other Asian ethnic groups increased with duration of residence, though modest evidence that it did so for people of Chinese ethnicity: those living in NZ for less than 10 years had a median rate ratio relative to the longest resident group of 0.73, 95% CI (0.49, 1.10) with only 7% of posterior rate ratios exceeding 1. Reflecting the increase in Chinese cancer mortality the Indian ethnic group, which had the lowest median cancer rate of around 52, had median posterior rate ratios (relative to people of Chinese ethnicity) that decreased in magnitude from 0.86 (DoR 0–9 years) to 0.61 (DoR 25+ years) and increased in significance of such that the credible interval in the longest DoR group (25+ years: 0.38, 0.97) excluded the null.

Results for CVD mortality were generally similar to those for all-cause mortality. For people of Indian ethnicity, CVD mortality rates were substantially higher than for other ethnicities at each DoR e.g., 92.3, 95% CI (65.6, 126.1) for those resident in NZ for 0–9 years, with a rate ratio (relative to the Chinese group with the same DoR) of 2.22, 95% CI (1.46, 3.40). For all ethnicities, CVD mortality rates for all ethnic groups living in NZ for less than 10 years were significantly less than those living in NZ for 25 years or more with median posterior rate ratios between 0.46 and 0.48. There was little evidence that CVD mortality was significantly different for the 10–24 year groups except for people of Indian ethnicity (rate ratio 0.71, 95% CI (0.41, 1.16)) with only about 9% of posterior rate ratios exceeding 1.

## Discussion

The findings of this study clearly indicate wide differences in all-cause mortality between Asian subgroups (our first research question). Indian Asians exhibited the highest standardised all-cause and CVD mortality rates of any subgroup. In contrast, Chinese and Other Asian cancer standardised mortality rates were significantly higher than those of Indian Asians. Increased rates of diabetes and cardiovascular disease for the Indian Asian group are probably important contributors to the increased all-cause and CVD mortality, and have been identified in New Zealand [50]–[55], in South Asian migrant populations in other countries [56]–[62], and in India [63]–[65]. The lower cancer mortality rates among Indians Asians are consistent with research on Asian Indians/Pakistanis in the US [66]; on South Asians in the UK [67] and in Canada [68]. This study cannot determine the reasons for this, but one contributing factor could be lower smoking prevalence among Indians as compared to Chinese in New Zealand [69].

The existence of differences in mortality between Asian subgroups has important implications from a health policy perspective which considers health disparities to be the same across all Asian groups and for all causes of death. Given the large and diverse groups of people the ‘Asian’ category encompasses, there has already been debate about the usefulness of ‘Asian’ as a single category [70]. Our findings support the contention that failure to take into account the heterogeneity of Asian subgroups risks masking areas of disparity and diversity of need [31], [70].

In response to our second research question (do overseas born Asian subgroups have a mortality rate advantage relative to their New Zealand-born counterparts of the same ethnicity, and does this relationship operate differently for different migrant subgroups), we found evidence of an all-cause mortality advantage for all OSB Asian subgroups over their NZB counterparts. Overseas born Asian subgroups have about two thirds of the mortality rate of NZB Asian counterparts and this relatively lower mortality rate among the OSB group showed little variation by Asian subgroup or cause of death: median rate ratios ranged between 0.63 and 0.71. Our results for an all-cause mortality advantage for OSB Asian subgroups are consistent with the conclusion of Hajat et al, 2010 [10] who also found evidence for a difference in all-cause mortality rates between OSB and NZB Asians using a combined 1996–1999 and 2001–2004 NZCMS dataset (though unlike the analyses reported here they did not test for significant temporal changes in mortality rate ratios between the two cohorts). Hajat et al (2010) also did not conduct analysis using ethnic-specific and cause-specific mortality.

The apparently lower mortality rate for the OSB Asian subgroups relative to their NZ born counterparts may result from a ‘healthy migrant effect’ (i.e., those migrating to the NZ are a much healthier group than those who remain in their countries of origin). This selection of healthy persons from the source country could be due to the tightening of immigration policies in the mid-1970s [41], resulting in stronger enforcement of the requirement that potential migrants undergo medical screening (direct health selection) [71]. Furthermore, changes in immigration policy from 1987 onwards favoured tertiary education, occupational skills, and wealth which may have added indirect health selection mechanisms to the immigration policy mix. All these factors cause Asian migrants to be healthier than the New Zealand born, contributing to the ‘healthy migrant effect’. Several studies of differing health outcomes have supported this hypothesis [26], [72]–[75].

Return migration of unhealthy migrants to their home country seems less likely to contribute to the observed migrant health advantage. Many factors could affect return migration: for example, distance from the ‘home’ country, ease of return, eligibility for superannuation and access to health care in the home country. Return migration is likely to be more common among Pacific people than among Asian migrants because of the shorter distance to the home nation and ease of return migration. Similarly, older migrants may not be eligible for NZ superannuation if they return to their home country. The quality of New Zealand health system may also be important: if care is perceived to be of a higher standard in New Zealand than in the country of origin the ‘unhealthy emigrant’ effect may be attenuated. Anecdotally, return migration for health reasons appears to be uncommon in the Asian population of New Zealand. However return migration does occur because of unemployment, which may in turn affect health and mortality, but this effect usually operates over a longer period than the inter-census timescale, and it may impact less on mortality rates.

Our third main research question (does the immigrant mortality advantage decline as duration of residence increases and is the decline the same for all OSB Asian ethnicities) is answered affirmatively for all-cause mortality with a steady increase in mortality for all ethnic groups as DoR increases. However, the amplitude of the advantage varies depending upon subgroup and cause of death. While there was evidence of significantly lower all-cause mortality rates for the OSB living 0–9 years and 10–24 years in NZ compared to longer-standing OSBs (25+ years), rate ratios were larger for people living in NZ between 10–24 years (around 0.8). There was no evidence of changes in cancer mortality with DoR for Indian and Other Asian groups though modest evidence of lower cancer mortality rates for OSB Chinese people living in NZ for 0–9 years compared to longer standing OSB (25+ years). However, Chinese, Indian and other Asian CVD mortality rates in the 0–9 DoR group were significantly less than for the 25+ year group.

Many explanations can be put forward for the increase in all-cause and CVD mortality of Asian immigrants with increasing time in New Zealand. Theories of acculturation [76], [77] would suggest that the replacement of healthier home country foods for fast-foods; the uptake in smoking, drinking alcohol, and other potentially harmful behaviours; the potential loss of social support networks; and the general stress of immigration may all contribute to the eventual loss of the health advantage [21], [76], [78]. However, this explanation may not be true for Asian migrants to New Zealand. For example, it is possible that the New Zealand health ‘environment’ may not have an adverse effect on the health of migrants: lower levels of pollution, opportunities for physical activity and education, and greater health service utilization are likely to result in improved rather than worsening health [5]. If so, acculturation would have a predominately positive affect on health. In general, the health advantage of the OSB will decrease, leading to an increasing mortality rate for migrants (relative to the NZB) over time, if acculturation is predominantly negative. Conversely the health advantage of the OSB will increase, leading to a decreasing mortality rate for migrants (relative to the NZB) over time, if acculturation is predominantly positive.

For Asian migrants, other factors may be important for the decline of health with increased duration of residence. The features which may have produced an advantaged health position pre-migration may not transfer to the new country. For example, income in New Zealand may be low (relative to the general NZ population) despite having a previously highly paid position in an Asian society. The structures of society are also different. For example, while holding a highly regarded position in an Asian society, a person’s place in the social hierarchy will likely have lowered following migration, perhaps permanently. This change of position may have future health consequences. Moreover, the erosion of a migrant health advantage can also be modified by discrimination: increasing years since migration may be associated with increasing (accumulated) exposure to discrimination [79] with the attendant negative consequences for health. All migrants are at some risk of experiencing discrimination because of their overseas born status, but characteristics such as visible minority status may place such migrant communities at additional risk [36]. There is some evidence from New Zealand that Asians have high self-reported experience of racism [80]. Similarly the Challenging Racism survey in Australia suggests that people of South Asian origin are also a highly discriminated ethnic group [81] in that country.

While these theories (selection, acculturation and discrimination) provide important frameworks within which the health inequalities between OSB and NZB can be considered, in reality they provide a rather simplistic perspective. For example, an overall decline in the health status of migrants is likely to be the result of the factors that begin well before migration and follow the migrating population, and possibly future generations, to the host country [29]. The history of migration, including changes in government migration policy over time, and the experience of migration can affect the future health of the migrants. For example, people migrating during the 1970s and earlier dominate mortality in the 25+ group. This ‘cohort’ is very different to those who migrated after the 1987 changes in migration policy, and consists of people who migrated several decades ago when there relatively few Asian migrants in New Zealand. They tended to come from poor village backgrounds, and if they were educated here very few made it to tertiary level. While their socio-economic status (SES) measured in the dataset used for this analysis may be moderate-high, the experience of lower SES during their early years may have influenced health outcomes. Thus, increased mortality rates may not be caused by negative acculturation or discrimination but by early life experiences. In short, the pathways and mechanisms by which migrant health changes over time occur are complex and require longitudinal data to address these issues. However, showing that the amplitude of health advantage varies depending upon subgroup, causes-of-death and duration of residence has opened the door to a potentially fruitful area of research for Asian migration.

### Limitations

One potential limitation relates to the possibility of residual linkage bias. Not all mortality records are liked to a census record in the NZCMS, but the use of linkage weights has been shown to sufficiently adjust for potential bias caused by SES and ethnicity. We should note the possibility, however, of residual linkage bias depending on birthplace and duration in NZ. Nevertheless, any such residual linkage bias would have to be unexpectedly large to change our conclusions regarding the marked mortality advantage for overseas born Asian subgroups (relative to NZ born counterparts), and the mortality advantage for the 0–9 year DoR group relative to the longest DoR group. Secondly, we have not attempted to separate the effects of duration of residence and age at migration which potentially impact the health of Asian migrants in different ways.

### Strengths

One of the main strengths of this study is that we explored Asian subgroup mortality – something which is hampered by the small number of deaths among subgroups and which in previous analyses did not allow study of these groups separately. Secondly, we conducted the analysis using all-cause and cause-specific mortality. Thirdly, we have further demonstrated the value of using hierarchical Bayesian methods for sparse data problems. We anticipate that the use of such methods will accelerate in the future, both within New Zealand and internationally, for a range of research questions including the monitoring and understanding of the health of ethnic minorities. Fourthly, our study used a high quality sample of sufficient size to see the differences in ethnic-specific mortality rates. The NZ census is conducted every 5 years and will continue to be linked to mortality records. Thus, research can continue to explore immigrant health as the population changes over time. Fifthly, for the nativity analysis we compared Asian subgroup immigrants to their NZB counterparts, whereas for the duration of residence analysis the reference group was migrants who had been in NZ the longest, increasing the internal validity of the study.

## Conclusion

This work has demonstrated that there is marked difference in mortality rates between Asian subgroups and between OSB and NZB Asian groups. Indian Asians exhibited the highest standardised all-cause mortality rates of any subgroup, followed by the other Asian and Chinese Asians. We found evidence of a mortality advantage (for all causes of death) for all OSB Asian subgroups over their NZB counterparts - an advantage that declines with the duration of residence in New Zealand. However, we have also shown that the health advantage of immigrant Asian subgroups varied depending upon subgroup ethnicity, cause of death, and duration of residence, reinforcing the need that these groups should be treated separately. Indian Asians had the lowest cancer mortality rates and highest CVD mortality rates. Aggregating these diverse and heterogeneous groups risks masking subgroup (and cause of death) differences in health outcomes and inappropriately targeting services and funds. The reasons for the apparently lower all-cause and CVD mortality rate for the 0–9 year DoR group are likely a result of a complex set of processes operating over time within the migrants’ social, political and cultural position in the host community, and simply attributing this to the negative aspect of acculturation is too simplistic. Future research on the exact mechanisms of the worsening of health with increased time spent in a host country would improve the understanding of the process and would assist the policy-makers and health planners.
